# Dynamics of place, boundary and object encoding in rat anterior claustrum

**DOI:** 10.3389/fnbeh.2015.00250

**Published:** 2015-10-14

**Authors:** Maciej M. Jankowski, Shane M. O’Mara

**Affiliations:** Institute of Neuroscience, Trinity CollegeDublin, Ireland

**Keywords:** claustrum, claustral function hypothesis, place cells, border cells, object cells, position, boundaries and landmark information

## Abstract

Discrete populations of brain cells signal differing types of spatial information. These “spatial cells” are largely confined to a closely-connected network of sites. We describe here, for the first time, cells in the anterior claustrum of the freely-moving rat encoding place, boundary and object information. This novel claustral spatial signal potentially directly modulates a wide variety of anterior cortical regions. We hypothesize that one of the functions of the claustrum is to provide information about body position, boundaries and landmark information, enabling dynamic control of behavior.

## Introduction

The claustrum of the mammalian brain is an anatomically-substantial but largely unexplored and uninvestigated structure (Edelstein and Denaro, 2004). In the rat, it is superior to the orbitofrontal cortex (rostrally) and approximately parallel to the insular cortex (laterally). It has a complex three-dimensional morphology across its longitudinal axis. Anatomical studies in the cat indicate an antero-posterior gradient of cortical connectivity in the claustrum: associational regions of cortex connect with anterior claustrum, and sensory areas of cortex connect with posterior claustrum (Olson and Graybiel, 1980; Pearson et al., 1982; Sadowski et al., 1997; Kowiański et al., 1998, 2001; Fernández-Miranda et al., 2008). Furthermore, dorsal claustrum projects to neocortical areas and ventral claustrum projects to subcortical or limbic structures (Olson and Graybiel, 1980; Kowiański et al., 1999). The limbic areas with which the claustrum communicates include many thalamic nuclei, the medial septal nuclei, amygdala, cingulate gyrus, subiculum, retrosplenial cortex and medial and lateral entorhinal cortex (Wilhite et al., 1986; Zhang et al., 2001; Majak et al., 2002; Park et al., 2012; Zingg et al., 2014). A monosynaptic claustral-entorhinal pathway in the rat was demonstrated via stimulation of claustral axon terminals in entorhinal cortex and recording of the antidromic single unit response in the claustrum (Wilhite et al., 1986). More recently, Zhang et al. (2013) reported retrogradely-labelled cells in the claustrum, which has very substantial unidirectional projections to the hippocampus (Witter et al., 1988). Studies in the hedgehog, rat, cat, tree-shrew, and the macaque all suggest that the claustrum projects to dorsal thalamic nuclei, striatum and hypothalamus (Mathur, 2014). Anatomical connectivity data suggest that claustrum has extensive direct and indirect connections with the entorhinal-hippocampal axis involved in spatial navigation, regions containing head direction, border, grid, place and object cells (O’Keefe and Dostrovsky, 1971; Taube et al., 1990; Hafting et al., 2005; Solstad et al., 2008; Park et al., 2012; Tsao et al., 2013). Thus, there is the possibility of functional interactions between the entorhinal-hippocampal neuraxis and claustrum.

The claustrum has been the subject of a limited degree of speculation regarding its potential functions. Crick and Koch (2005) speculate it plays a key role in the generation of consciousness in the mammalian brain. Smythies et al. (2012) and Smith et al. (2012) suggest it plays a key role in organizing and synchronizing cortical activity and is important for coordinating motor behaviors involved in redirecting spatial attention. Data testing these views are currently unavailable. One study, conducted in the head fixed and immobilized non-human primate, found that single cell, unimodal sensory responses predominate within the claustrum when using naturalistic stimuli (Remedios et al., 2010). More recently, Remedios et al. (2014) reported that claustral neurons in rhesus monkeys respond to target sounds embedded in a noisy background. The single trial responses of individual neurons suggested that claustral cells may detect and reflect the occurrence of a change in the auditory scene. The authors suggest that the claustral neurons may be involved in detection of new events critical for behavior and survival, as suddenly appearing objects may require rapid and coordinated reactions. To our knowledge, no *in vivo* neurophysiological recordings have ever been conducted in the claustrum in the freely-moving, awake, behaving animal. Here, we describe, in the freely-moving rat, recordings of multiple single neurons in the anterior claustrum. Our data suggest, unexpectedly, the presence of cells that are responsive to the position in space of the animal, to boundaries enclosing the environment and to the presence of objects in the environment.

## Results

### Phenotypes, Numbers and Percentages of Cells Recorded in Anterior Claustrum

In total, 874 well-isolated units recorded in 4 rats during 348 recording sessions were assigned to the anterior claustrum, after post-mortem histological verification. The total number of cells was derived after sorting of 2952 clusters of spikes. Units were sometimes recorded for more than for 1 day, despite lowering of the electrodes. In those cases, during the final spike sorting, cells were monitored on the relevant tetrodes from day-to-day; for analysis, only clean recordings with the biggest sample size and spikes of the highest amplitude were chosen. Particular care was taken to exclude seemingly-related samples from analysis to avoid inadvertent double-counting of cells. During spike sorting, signals from each cell were carefully followed from first appearance to complete loss, in order to avoid overestimation in the cell counts. Additionally, ambiguous sessions were rejected. We were conservative during spike sorting; we followed each cell individually from first appearance to loss, assigning different number of clusters to each of the cells which were present (from 1–14 days). Variations in the numbers of cells between animals are caused by the differing locations of electrodes in implanted animals. Higher cell counts were observed in animals in which bundles of eight tetrodes were implanted in the more caudal part of anterior claustrum (just before the beginning of the striatum on the coronal sections). Smaller counts were found in rats implanted in the more difficult to access rostral portion of anterior claustrum, where the claustrum is significantly smaller. Electrodes were implanted at an angle of 13° to extend the length of the electrode track through the claustrum at locations corresponding to the following coronal sections: +2.70 mm to bregma in Paxinos and Watson (1998), or sections +3.24 and +3.00 mm in Paxinos and Watson (2006; there are discrepancies in the thickness of claustrum at different antero-posterior coordinates between these differing versions of atlases and rat strains). Electrodes in two animals with high counts of cells passed through the central portion of the caudal anterior claustrum. With 32 tetrodes in four animals, we recorded 2952 clusters, which provided 874 cells after removing multiple recording sessions for the same cells. Typically, between one and seven clusters were recorded from each tetrode during recording sessions. Chronic electrophysiological recordings of differing cell types in a freely-moving animal are challenging. Cells are held for an unpredictable amount of time, making it difficult to conduct all manipulations for all cells. Moreover, rats are food restricted and can perform only up to 4 recording sessions per day (usually 16–20 min during which they run distances between 140–250 m) to fulfill the criteria for exploration density. Therefore, not all manipulations were performed for all cells.

Cells were classified according to their spatial and temporal properties as follows: 38 place cells (4.3%); 23 boundary cells (2.6%); 48 object cells (5.5%); 25 other spatially-tuned or object-like cells (2.8%; Table 1). Place cells were observed in three rats, object cells in two rats and boundary cells in two rats. There were 96 bursting, high firing rate cells (10.9%); this category was subdivided based on firing frequencies into two subclasses: a subgroup of 59 bursting cells with a maximum firing frequency between 20 and 30 Hz (6.7%), and a subgroup 37 bursting cells with a maximum firing frequency of >30 Hz (4.2%) at any location in the open field environment (Table 2). We also recorded 37 weakly-theta-modulated cells (4.2%); 24 theta-modulated cells (2.7%); four non-bursting fast-firing cells with a maximum firing frequency of >30 Hz (0.45%). A total of 596 cells (68%) in all rats were classified as unidentified low firing units—cells that did not exhibit any particular temporal or spatial properties.

**Table 1:** **Electrophysiological classification of claustral units with spatial, boundary or object properties (mean ± SEM)**.

Cell types	Place cells	Boundary cells	Object cells	Other spatially-tuned or object-like cells
N (%)	38 (4.3%)	23 (2.6%)	48 (5.5%)	25 (2.8%)
Mean spike amplitude [μV]	172.2 ± 28.0	113.3 ± 8.90	108.4 ± 3.72	132.7 ± 11.4
Mean spike width [μs]	161.9 ± 6.21	148.0 ± 7.59	178.0 ± 3.46	153.1 ± 8.30
Mean firing frequency [Hz]	1.02 ± 0.18	1.01 ± 0.19	0.79 ± 0.11	1.40 ± 0.37
Spatial coherence	0.52 ± 0.02	0.40 ± 0.03	0.51 ± 0.03	0.45 ± 0.04
Spatial Information Content (Skaggs)	1.69 ± 0.10	1.50 ± 0.12	1.85 ± 0.10	1.72 ± 0.29

Summary statistics for mean spike amplitude, mean spike width, mean firing frequency, spatial coherence and spatial Information content (Skaggs) for all spikes (mean ± SEM) for the place, boundary and object cells recorded in anterior claustrum. One-way ANOVA followed by Tukey’s test revealed that: mean spike amplitude is significantly bigger in place cells than in object cells OC (*p* < 0.05); mean spike width in object and place cells is significantly bigger than in boundary cells (*p* < 0.01) and in object cells mean spike width is significantly bigger than in other spatially-tuned or object-like cells (*p* < 0.05); spatial coherence is significantly bigger in place cells than in boundary cells (*p* < 0.05).

**Table 2:** **Electrophysiological classification of claustral bursting fast-firing cells (mean ± SEM)**.

Cell types	Bursting cells—whole not divided population	Bursting cells with maximum firing frequency 20–30 Hz	Bursting cells with maximum firing frequency >30 Hz
N (%)	96 (10.9%)	59 (6.7%)	37 (4.2%)
Mean spike amplitude [μV]	179.51 ± 7.50	179.2 ± 9.80	180.0 ± 11.7
Mean spike width [μs]	109.78 ± 2.43	110.9 ± 2.97	108.0 ± 4.21
Mean firing frequency [Hz]	22.48 ± 0.74	18.2 ± 0.37	29.3 ± 1.14****
Maximum firing frequency [Hz]	30.13 ± 0.91	24.6 ± 0.35	38.9 ± 1.34****
Spatial coherence	0.68 ± 0.008	0.65 ± 0.01	0.72 ± 0.01****
Spatial information content (Skaggs)	0.07 ± 0.003	0.08 ± 0.005	0.05 ± 0.003****

Summary statistics for mean spike amplitude, mean spike width, mean firing frequency, maximum firing frequency, spatial coherence and spatial Information content (Skaggs) (mean ± SEM) for all bursting cells and the two categories of cells with maximum firing frequency at any location in open field environment in the range 20–30 Hz and bursting cells with maximum firing frequency >30 Hz recorded in anterior claustrum. In both groups cells with tendency to bi- or even tri-phasic mode of firing were observed. A two-tailed Student *t*-test revealed that those two categories differ significantly in: mean firing frequency (****p < 0.0001); maximum firing frequency (****p < 0.0001); spatial coherence (****p < 0.0001); and spatial Information content (****p < 0.0001).

### Place Cells are Present in the Claustrum During Light-Dark-Light Recordings

**Figure 1:**
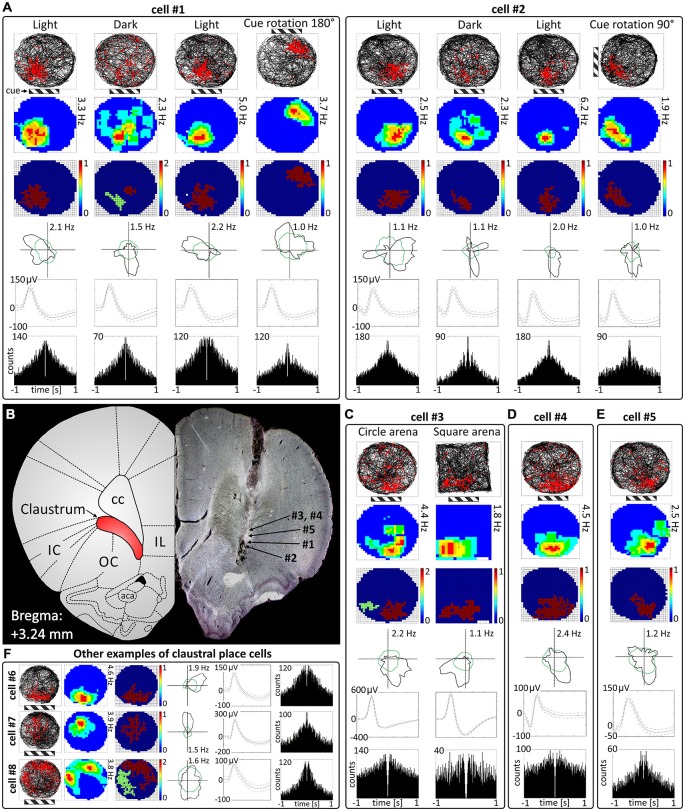
**Place cells in rat anterior claustrum. (A)** Examples of two spatially-tuned claustral cells recorded over consecutive sessions with changes in environmental conditions. Each cell was recorded in the light, darkness, light and after rotation of visual cue in the light. The pattern of response suggests that activity of claustral place cells is anchored to visual cues (the location of main cue—cue card with black and white stripes is marked on the figure by striped rectangle). For each recording session the following are presented in columns: path of the animal recorded during 20-min session with superimposed firing activity of the unit; firing intensity map with a maximum firing frequency; place map; polar plot; spike waveform and autocorrelation ±1000 ms (those parameters are presented in similar layout in **C–E**). **(B)** Estimated locations of spatially-tuned cells presented in this figure reconstructed on histological specimen. On the left side of the histological slide, a schematic anatomical figure with borderlines between structures for similar antero-posterior coordinates is presented. **(C)** Claustral place cells form place fields in environments of different geometrical shapes when tested after transitions from a circular to square environments in the light. **(D,E)** Example of two more place cells recorded in the light. The location of place fields was similar in majority of cells suggesting that their activity may be strongly anchored to the visual and possibly other cues in experimental room. **(F)** Examples of three place cells with different locations of place fields in the experimental arena. Place fields located in other parts of experimental arena than those formed by cells #1—#6 were less prevalent (like in cells #7 and #8). Cell #7 exhibited both spatial tuning and directional tuning. For each cell in rows are presented: path of the animal with superimposed firing activity, firing intensity map, place map, polar plot, spike waveform and autocorrelation ± 1000 ms. Abbreviations: aca, anterior commissure, anterior part; cc, corpus callosum; IC, insular cortex; IL, infralimbic cortex; OC, orbital cortex.

**Figure 2:**
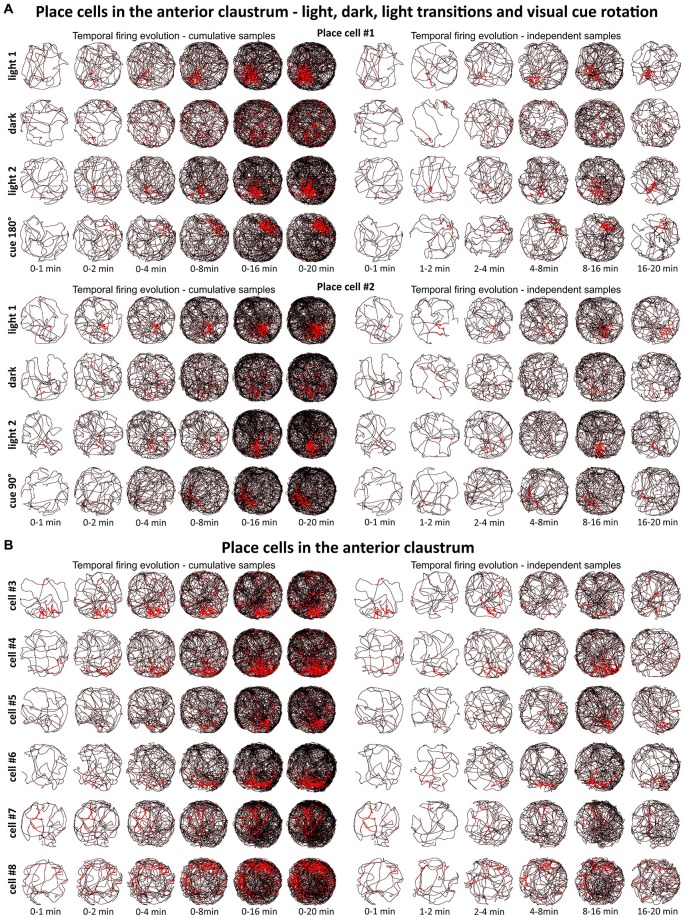
**Temporal evolution of firing of claustral place cells. (A)** Temporal evolution of firing for place cells #1 and #2. Each cell was recorded in the light, darkness, light and after rotation of visual cue in the light. **(B)** Temporal evolution of firing for place cells #3—#8. For each cell is presented one recording session performed in the light. In both panels **(A,B)**, on the left six samples of cumulative time intervals (0–1, 0–2, 0–4, 0–8, 0–16 and 0–20 min) and on the right six samples of independent time intervals (0–1, 1–2, 2–4, 4–8, 8–16 and 16–20 min) are presented for each cell/condition in the rows. The visual cue was located in the same place as in Figure 1 in baseline conditions.

Cells exhibiting the characteristic phenotype of classical hippocampal place cells were found in the claustrum (examples of these cells and their estimated location on a histological specimen are presented in Figure 1). In total, 38 place cells (4.3%) were recorded in the claustrum. These cells show sharply-defined, location-specific firing during recordings in the light and the dark. Lighting conditions were systematically varied across foraging sessions for eight cells (and for some cells more than once). The animal foraged during light-dark-light sessions (for either 16 or 20 min). Rotation of the visual cue by 90° or 180° induced repositioning of firing fields which shifted by similar angle (Figure 1A). Such changes in the firing activity of claustral place cells in relation to environmental manipulations suggest that their activity might be anchored to visual cues. In the dark, place fields seem visually less sharply defined when tested across light dark-light transitions (Figure 1A). However, no statistically significant differences were found between light-dark-light conditions in mean firing rate (light_1: 1.20 ± 0.56 Hz vs. dark: 0.97 ± 0.79 Hz vs. light_2: 1.63 ± 1.06 Hz, light_1 vs. dark *t*_(7)_ = 0.86, dark vs. light_2 *t*_(7)_ = 2.22, light_1 vs. light_2 *t*_(7)_ = 0.86), maximum firing frequency (light_1: 4.15 ± 1.38 Hz vs. dark: 3.37 ± 2.15 Hz vs. light_2: 5.47 ± 2.39 Hz, light_1 vs. dark *t*_(7)_ = 0.84, dark vs. light_2 *t*_(7)_ = 2.89, light_1 vs. light_2 *t*_(7)_ = 1.16) and spatial coherence (light_1: 0.56 ± 0.06 vs. dark: 0.35 ± 0.08 vs. light_2: 0.49 ± 0.08, light_1 vs. dark *t*_(7)_ = 2.79, dark vs. light_2 *t*_(7)_ = 2.10, light_1 vs. light_2 *t*_(7)_ = 1.35, mean ± SEM, multiple comparisons with two-tailed *t* test for paired two samples for means with Bonferroni correction). Measured parameters varied strongly between the cells, and there were more and less active neurons with differing levels of spontaneous baseline activity. Interestingly, spatial information content (Skaggs) was significantly higher in the darkness (2.84 ± 0.37) than in the first, preceding recordings in the light (1.88 ± 0.26, *t*_(7)_ = 4.63, *p* < 0.01, two-tailed *t* test for paired two samples for means with Bonferroni correction). There were no significant differences in spatial information content (Skaggs) between recordings in the dark (dark: 2.84 ± 0.37) vs. following recording in the light (light_2: 2.26 ± 0.38, *t*_(7)_ = 2.31) as well as between first (light_1: 1.88 ± 0.26) and the final recording in the light (light_2: 2.26 ± 0.38, *t*_(7)_ = 1.97, mean ± SEM, two-tailed *t* test for paired two samples for means with Bonferroni correction). Claustral place cells form place fields in similar locations in environments of different geometrical shapes as tested in subsequent recordings performed in circular and square arenas in the light (Figure 1C). A further observation supporting the idea that the firing activity of recorded cells might be anchored to visual cues is that the majority of cells formed place fields located in the same part of circular arena in baseline conditions in which visual cue was always present in the same location (Figures 1A–F, cells #1–6). However, we also recorded cells that formed place fields in other locations, but they were less prevalent (Figure 1F, cells #7 and #8). Some cells (e.g., cell #7 of Figure 1F) showing spatial tuning also showed some directional tuning. Directional tuning in those cells might be caused by the particular orientation of the animal’s head when it attends to particular visual stimuli in the experimental environment (but this possibility remains to be tested in future experiments). Support for this possibility would reinforce the hypothesis that claustral cells might be anchored to visual stimuli. Finally, the activity of claustral place cells remains stable and constant across the duration of the recording session (Figure 2).

### Place Cells Fire from the First Exposure to an Environment

We tested time of onset of first firing for our claustral place cell samples. A delayed onset might suggest (Hok et al., 2012) that there is either instability of place representation or an online remapping of place representation is occurring. Figure 2 depicts the temporal evolution of spatial firing in the claustrum for cumulative samples (left-hand columns) and independent time-binned samples (right-hand columns). Spatial activity is present from the first minute of exposure to the environment, indicating a rapid and near-instantaneous expression of place representation by these claustral place cells.

### Claustral Place Cells do not React to the Presence of Objects in the Arena

Discrete objects introduced into the environment do not significantly change the firing properties of claustral place cells, which maintain the previous location of their place fields despite the presence of the object. A representative example of a claustral place cell recorded with and without the presence of the object is shown in Figure 8A. Twelve place cells recorded in sessions with and without the object showed no statistically significant differences in mean firing rate (no object: 0.78 ± 0.13 Hz vs. object: 0.57 ± 0.18 Hz, *t*_(11)_ = 0.59), maximum firing frequency (no object: 3.62 ± 0.60 Hz vs. object: 2.57 ± 0.74 Hz, *t*_(11)_ = 1.98), spatial coherence (no object: 0.57 ± 0.03 vs. object: 0.49 ± 0.07, *t*_(11)_ = 1.72) and spatial information content (Skaggs, no object: 1.85 ± 0.19 Hz vs. object: 2.15 ± 0.37 Hz, *t*_(11)_ = 0.79, mean ± SEM, two-tailed *t* test for paired two samples for means).

### Spatially-Tuned Cells Signaling the Physical Borders of the Environment are Present in the Claustrum

**Figure 3:**
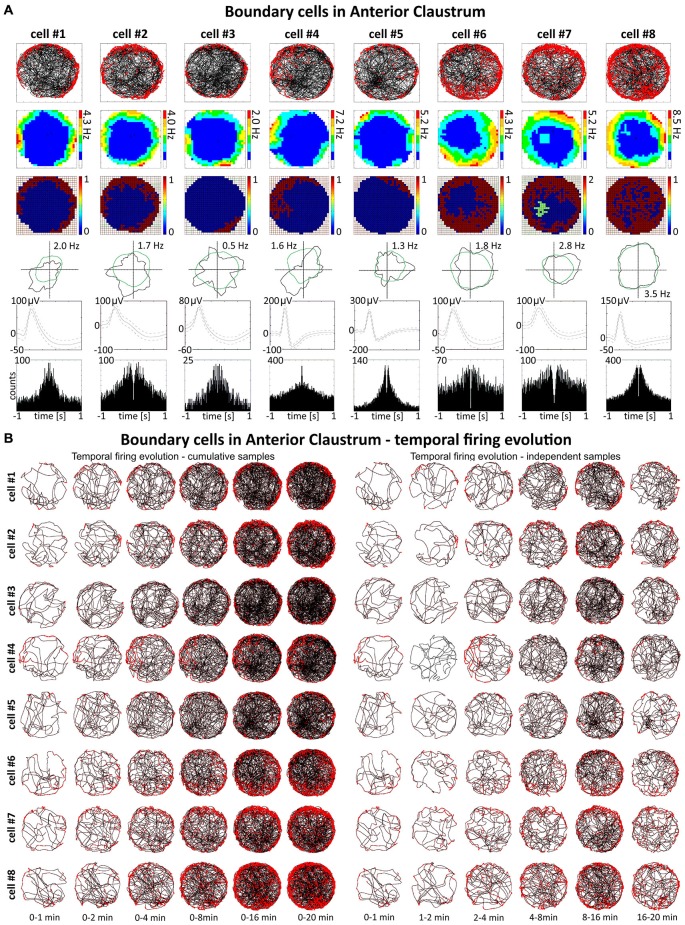
**Boundary/border cells in rat anterior claustrum. (A)** Cells that react with increased firing activity in response to the physical geometric borders of the environment. For each cell in columns are shown: path of the animal recorded during a 20-min session with superimposed firing activity; firing intensity map with a maximum firing frequency; place map; polar plot; spike waveform and autocorrelation ± 1000 ms. The visual cue was located in the same place as in Figure 1 in baseline conditions. **(B)** Temporal evolution of firing of boundary cells shown in **(A)**. On the left, six samples of cumulative time intervals, and on the right, six samples of independent time intervals are presented in rows for each cell recorded during a 20 min session. Boundary cells responded through the whole session from the beginning to the end (an exception is cell #4 which did not respond during second minute of recording).

Some spatially-tuned claustral units fired when the rat was close to a physically-defined boundary in the circular arena, potentially signaling the presence of a border or environmental perimeter. We assigned such cells to the category “boundary cells”, all cells that formed longitudinal firing fields adjacent to the walls of the circular arena. Figure 3A provides examples of the firing properties of these cells. In total 23 boundary cells (2.6%) were recorded in the claustrum. The firing activity of claustral boundary cells remains stable and constant across the duration of the recording session. Figure 3B depicts the temporal evolution of spatial firing in the claustrum for cumulative samples (left-hand columns) and independent time-binned samples (right-hand columns). Firing is present in the first minute of exposure to the environment, indicating a rapid or near-instantaneous expression of boundary representation by those claustral boundary cells. Boundary cells are active in the light and darkness and preserve their firing properties in the light and dark. No statistically significant differences were found between lighting conditions in mean firing rate (light: 1.93 ± 0.55 Hz vs. dark: 1.30 ± 0.76 Hz, *t*_(3)_ = 0.43), maximum firing frequency (light: 6.52 ± 0.91 Hz vs. dark: 4.35 ± 1.65 Hz, *t*_(3)_ = 0.76), spatial coherence (light: 0.46 ± 0.08 vs. dark: 0.46 ± 0.07, *t*_(3)_ = 0.005) and spatial information content (Skaggs, light: 1.31 ± 0.45 vs. dark: 1.12 ± 0.37, *t*_(3)_ = 1.64, mean ± SEM, two-tailed *t* test for paired two samples for means, results from four representative cells). We also tested the effects of introducing an object into the arena on the firing properties of claustral border cells (Figure 8D). No statistically significant differences were found in mean firing rate (no object: 0.75 ± 0.19 Hz vs. object: 0.87 ± 0.25 Hz, *t*_(4)_ = 1.04), maximum firing frequency (no object: 3.63 ± 0.75 Hz vs. object: 4.12 ± 0.88 Hz, *t*_(4)_ = 1.59), spatial coherence (no object: 0.45 ± 0.04 vs. object: 0.43 ± 0.05, *t*_(4)_ = 0.28) and spatial information content (Skaggs, no object: 1.86 ± 0.33 vs. object: 1.62 ± 0.19, *t*_(4)_ = 0.98, mean ± SEM, two-tailed *t* test for paired two samples for means, results from five representative cells). Cue rotations, recordings in environments of different shape and presence of multiple objects also did not change firing properties of claustral boundary cells. Thus, the presenting or manipulating single or multiple objects in the environment does not affect firing properties of claustral boundary cells (Figure 8D).

### Object Cells are Present in the Claustrum

**Figure 4:**
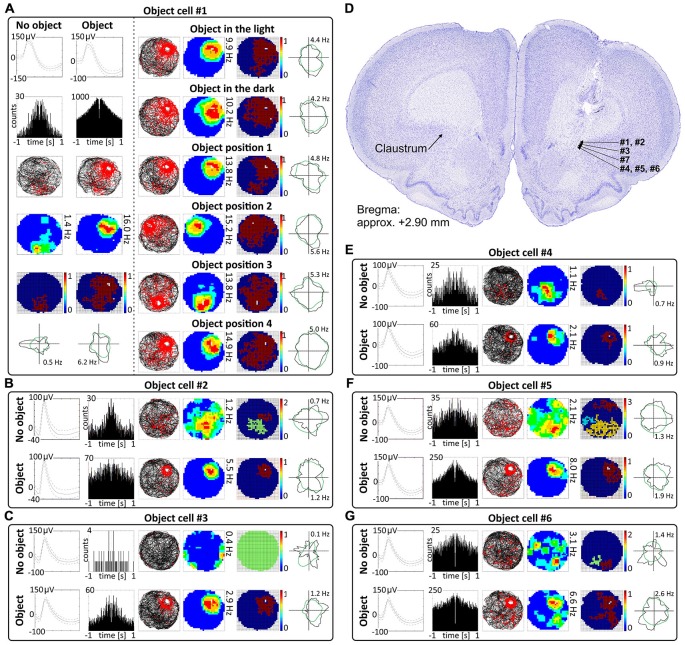
**Object cells in rat anterior claustrum. (A)** Claustral object cells responded to the presentation of an object (glass bottle) with increased firing activity; a firing field was created in the proximity to the object. The visual cue was located in the same place as in Figure 1 in baseline conditions. **(A)** On the left, two consecutive recording sessions of an object cell with and without presentation of the object. For each recording session presented in columns are: spike waveform; autocorrelation ± 1000 ms; path of the animal recorded during 20 min session with superimposed firing activity; firing intensity map with maximum firing frequency; place map and polar plot. **(A)** On the right, recordings performed in the light, darkness and after changes of object’s position during consecutive recording sessions in the light. For each session in rows are shown: path of the animal; firing intensity map; place map and polar plot. **(B,C,E–G)** Examples of other object cells recorded in anterior claustrum. Recording sessions were performed in the light with and without presentation of the object. Cells expressed varied spontaneous firing rates and varied increases in firing activity in response to the object (e.g., cell #1 vs. cell #3). For each session, in rows, are shown: spike waveform; autocorrelation ± 1000 ms; path of the animal with superimposed firing activity; firing intensity map; place map and polar plot. **(D)** Estimated location of object cells presented in this figure reconstructed on a representative histological specimen.

**Figure 5:**
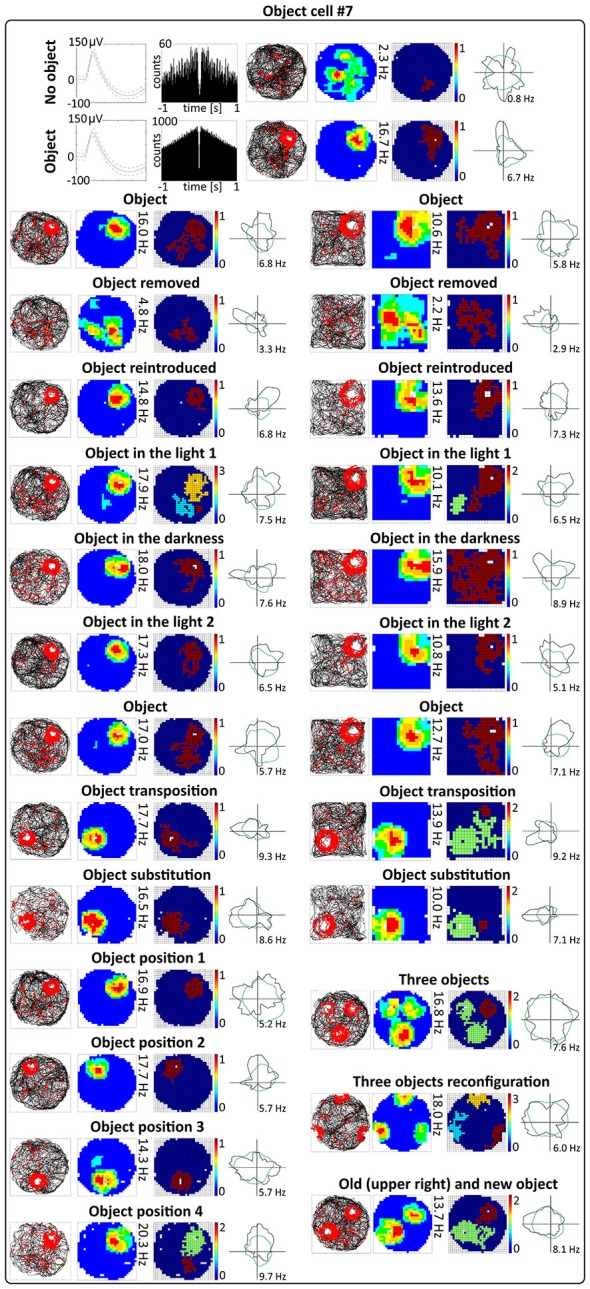
**Environmental manipulations for a representative claustral object cell.** Top: two consecutive recording sessions with and without presentation of the object (glass bottle). For each recording session in rows are presented: spike waveform: autocorrelation ± 1000 ms; path of the animal recorded during 20 min session with superimposed firing activity: firing intensity map with a maximum firing frequency, place map and polar plot. Bottom, recording sessions with environmental manipulations. The visual cue was located in the same place as in Figure 1 in baseline conditions. On the left, sets of consecutive recording sessions in circular arena with following transitions: object–no object–object; light–dark–light; object–object transposition–object substitution (substitution with object of different shape, color and material—painted wooden brick); object in position 1–2–3–4. On the right, consecutive recording sessions in the square arena with the following transitions: object–no object –object; light–dark–light; object–object transposition–object substitution. Below on the right, samples of recordings with presentation of three objects in two different spatial configurations and with simultaneous presentation of old and new object (plastic object presented to the animal for the first time). For all transitions in rows are shown: path of the animal; firing intensity map; place map and polar plot.

Cells that responded to the presence of discrete objects with increased firing activity only in the vicinity of the object were classified as object cells (See Supplementary Video 1). Those cells formed place fields around the objects, and their firing activity was subjected to the same analyses as claustral place cells, in order to compare these two spatially-tuned cell types. In total, 48 object cells (5.5%) were recorded in the claustrum. A sample of claustral object cells recorded before and after presentation of the object is shown in Figures 4 and 5 (Figure 4, cells #1–#6 and Figure 5; cell #7). Histological verification confirmed that these object cells were recorded in the anterior claustrum (Figure 4D). The majority of object cells had low spontaneous firing activity when the object was not present in the environment. Some cells were almost silent, and others had a higher spontaneous firing rate (maximum firing frequency ranged from 0.28–5.5 Hz). When an object (glass bottle with a textured surface) was placed in the environment, the firing activity of object cells significantly increased in the vicinity of the object, but responsivity to the object differed between cells (maximum firing frequency ranged from 0.46–16.74 Hz). Placement of the object significantly increased the maximum firing frequency (no object: 1.79 ± 0.32 Hz vs. object: 5.04 ± 1.01 Hz, *t*_(20)_ = 3.54, *p* < 0.01) and mean firing rate (no object: 0.48 ± 0.09 Hz vs. object: 1.14 ± 0.21 Hz, *t*_(20)_ = 3.38, mean ± SEM, two-tailed *t* test for paired two samples for means, *p* < 0.01). We also found a highly significant increase in spatial coherence in response to the object (no object: 0.30 ± 0.03 vs. object: 0.59 ± 0.04, *t*_(20)_ = 6.63, mean ± SEM, two-tailed *t* test for paired two samples for means, *p* < 0.0001) while spatial information content (Skaggs) was unaltered (no object: 1.96 ± 0.19 vs. object: 1.78 ± 0.13, *t*_(20)_ = 3.54, mean ± SEM, two-tailed *t* test for paired two samples for means).

### Changing Visual Conditions from Light to Dark to Light does not Affect Claustral Object Cells

Claustral object cells show increased firing activity around the object (a glass bottle with a textured surface) across changes in lighting conditions. The animals foraged during light-dark-light sessions in a circular arena (for 16 or 20 min). Recordings performed in the light and darkness showed that cells respond to objects in both conditions, and that their activity did not change significantly between light and dark conditions (Figures 4A, 5). No statistically significant differences were found between lighting conditions in mean firing rate (light_1: 1.28 ± 0.28 Hz vs. dark: 1.07 ± 0.24 Hz vs. light_2: 1.06 ± 0.22 Hz, light_1 vs. dark *t*_(13)_ = 1.72, dark vs. light_2 *t*_(13)_ = 0.12, light_1 vs. light_2 *t*_(13)_ = 1.92), maximum firing frequency (light_1: 5.76 ± 1.25 Hz vs. dark: 4.88 ± 1.10 Hz vs. light_2: 4.99 ± 1.08 Hz, light_1 vs. dark *t*_(13)_ = 1.83, dark vs. light_2 *t*_(13)_ = 0.29, light_1 vs. light_2 *t*_(13)_ = 1.40), spatial coherence (light_1: 0.63 ± 0.04 vs. dark: 0.60 ± 0.04 vs. light_2: 0.60 ± 0.04, light_1 vs. dark *t*_(13)_ = 1.29, dark vs. light_2 *t*_(13)_ = 0.09, light_1 vs. light_2 *t*_(13)_ = 0.78) and spatial information content (Skaggs, light_1: 1.86 ± 0.16 vs. dark: 1.79 ± 0.16 vs. light_2: 1.86 ± 0.18, light_1 vs. dark *t*_(13)_ = 0.63, dark vs. light_2 *t*_(13)_ = 0.72, light_1 vs. light_2 *t*_(13)_ = 0.01, mean ± SEM, multiple comparisons with two-tailed *t* test for paired two samples for means, results from 14 representative cells). Lighting conditions were systematically varied across foraging sessions for 16 cells (and for some cells more than once). Lighting conditions were also changed in the environments of different shape (circular vs. square arena) and firing activity of object cells was not affected significantly by those manipulations (Figure 5).

### Activity of Object Cells is Tightly Coupled to the Object in Circular and Square Arenas

**Figure 6:**
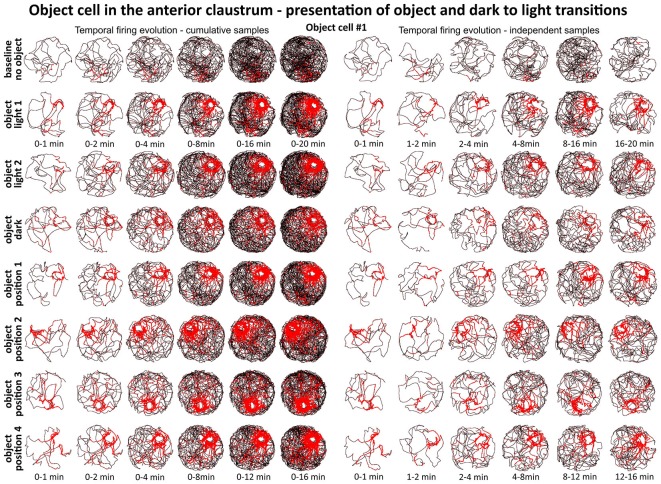
**Temporal evolution of firing of claustral object cell #1 from Figure 3.** On the left, six samples of cumulative time intervals, and on the right, six samples of independent time intervals presented in rows for recordings in each environmental condition. Sessions were either 16 or 20 min long. The object cell responded through the whole session from the beginning until the end. The visual cue was located in the same place as in Figure 1 in baseline conditions.

Activity of claustral object cells is tightly coupled to the object (a glass bottle with a textured surface). They follow the object when it is moved within the environment, and do not leave traces of firing at previous object locations on subsequent recording sessions. Firing fields are rapidly formed at the new object location. Firing in response to objects presented at different positions was tested using two protocols. First, during four consecutive recording sessions performed for four representative cells, the object was placed in three different locations in the arena at the same location on the first and last session (positions 1–2–3–4 in the corners of an equilateral triangle, recordings 16 or 20 min long, Figures 4A, 5). Object cells responded to the object presented at all tested locations in a similar fashion. For differing positions of the object, there were no statistically significant differences in mean firing rate (position_1: 2.08 ± 0.80 Hz, position_2: 1.96 ± 0.78 Hz, position_3: 2.15 ± 0.86 Hz, position_4: 2.58 ± 1.10 Hz, 1 vs. 2 *t*_(3)_ = 0.43, 1 vs. 3 *t*_(3)_ = 0.51, 1 vs. 4 *t*_(3)_ = 1.60, 2 vs. 3 *t*_(3)_ = 1.02, 2 vs. 4 *t*_(3)_ = 1.22, 3 vs. 4 *t*_(3)_ = 1.33), maximum firing frequency (position_1: 9.33 ± 3.88 Hz, position_2: 10.07 ± 4.20 Hz, position_3: 8.25 ± 3.42 Hz, position_4: 10.11 ± 4.54 Hz, 1 vs. 2 *t*_(3)_ = 1.60, 1 vs. 3 *t*_(3)_ = 1.93, 1 vs. 4 *t*_(3)_ = 0.85, 2 vs. 3 *t*_(3)_ = 2.16, 2 vs. 4 *t*_(3)_ = 0.04, 3 vs. 4 *t*_(3)_ = 1.30), spatial coherence (position_1: 0.69 ± 0.09, position_2: 0.71 ± 0.07, position_3: 0.66 ± 0.09, position_4: 0.64 ± 0.10, 1 vs. 2 *t*_(3)_ = 0.44, 1 vs. 3 *t*_(3)_ = 1.88, 1 vs. 4 *t*_(3)_ = 1.20, 2 vs. 3 *t*_(3)_ = 0.99, 2 vs. 4 *t*_(3)_ = 0.91, 3 vs. 4 *t*_(3)_ = 0.70) and spatial information content (Skaggs, position_1: 1.59 ± 0.10, position_2: 1.68 ± 0.24, position_3: 1.50 ± 0.18, position_4: 1.38 ± 0.13, 1 vs. 2 *t*_(3)_ = 0.54, 1 vs. 3 *t*_(3)_ = 0.57, 1 vs. 4 *t*_(3)_ = 1.76, 2 vs. 3 *t*_(3)_ = 1.64, 2 vs. 4 *t*_(3)_ = 2.04, 3 vs. 4 *t*_(3)_ = 2.42, mean ± SEM, multiple comparisons with two-tailed *t* test for paired two samples for means with Bonferroni correction). Firing activity of object cells was present from the first minute of exposure to the environment with the discrete object, indicating that there is a rapid or near-instantaneous expression of object representation by these claustral cells. Figure 6 depicts the temporal evolution of firing of a representative object cell in response to object presented in positions 1–4 on subsequent recording sessions for cumulative samples (left-hand columns) and independent time-binned samples (right-hand columns).

The other object protocol involved three consecutive recording sessions (object–object transposition–object substitution), and this object protocol also confirmed that object cells follow the object without leaving any trace of activity after in the previous object position. In this protocol, the object was moved to the opposite quadrant of the circular arena. The effect of object transposition was measured for 17 cells during 16 or 20 min long sessions (a representative example is shown on Figure 5). There were no statistically significant differences between the previous and new position of the object in mean firing rate (object baseline: 1.06 ± 0.17 Hz vs. object transposition: 0.96 ± 0.22 Hz, *t*_(16)_ = 0.80), maximum firing frequency (object baseline: 4.54 ± 0.89 Hz vs. object transposition: 4.39 ± 1.09 Hz, *t*_(16)_ = 0.31), spatial coherence (object baseline: 0.55 ± 0.04 vs. object transposition: 0.45 ± 0.05, *t*_(16)_ = 2.09), and spatial information content (Skaggs, object baseline: 1.69 ± 0.11 vs. object transposition: 1.81 ± 0.13, *t*_(16)_ = 1.13, mean ± SEM, two-tailed *t* test for paired two samples for means). Object transposition was tested in circular as well as in square arena and object cells behaved similarly in both environments.

### Object Substitution does not Affect Claustral Object Cells

When one object is substituted by another object of different shape, color and material, claustral cells react to it in similar ways as to the previous object, suggesting that claustral object cells dynamically code the position of the object in space and not its unique features as an object. We found that when one object (a transparent glass bottle) is substituted with an object of different material, texture, shape and color (a colorful wooden flower), it will still induce a similar response in claustral object cells. Object substitution was measured for 16 cells during 16 or 20 min long sessions (an example is shown on Figure 5). Object cells did not show statistically significant differences in mean firing rate (object: 1.03 ± 0.19 Hz vs. object substitution: 1.06 ± 0.27 Hz, *t*_(15)_ = 0.19), maximum firing frequency (object: 4.56 ± 0.94 Hz vs. object substitution: 3.98 ± 0.96 Hz, *t*_(15)_ = 1.69), spatial coherence (object: 0.54 ± 0.05 vs. object substitution: 0.49 ± 0.06, *t*_(15)_ = 0.90), and spatial information content (Skaggs, object: 1.79 ± 0.13 vs. object substitution: 1.62 ± 0.15, *t*_(15)_ = 1.28, mean ± SEM, two-tailed *t* test for paired two samples for means) in response to those two distinct objects. Substitution was tested in circular and square arena and in both environments object cells behaved similarly.

### Changing the Geometrical Shape of the Environment does not Affect Claustral Object Cells

Changing the geometrical shape of the environment from circle to square did not affect the responses of object cells to the presentation of objects (recordings performed for seven representative cells). Furthermore, responses were not changed in different lighting conditions (Figure 5). There were no statistically significant differences between object cells recorded in the light in circular vs. square arenas for mean firing rate (circle_light: 1.47 ± 0.79 Hz, square_light: 1.39 ± 0.42 Hz, *t*_(3)_ = 0.19), max firing frequency (circle_light: 6.49 ± 3.68 Hz, square_light: 3.79 ± 1.78 Hz, *t*_(3)_ = 1.41) and spatial coherence (circle_light: 0.52 ± 0.15, square_light: 0.43 ± 0.12, *t*_(3)_ = 0.73). Spatial information content was slightly lower in the square arena (Skaggs, circle_light: 1.52 ± 0.10, square_light: 0.82 ± 0.21, *t*_(3)_ = 2.35, *p* < 0.05, mean ± SEM, two-tailed *t* test for paired two samples for means, data from representative four cells). We did not find statistically significant differences between object cells recorded in circular vs. square arenas in the darkness in mean firing rate (circle_dark: 1.29 ± 0.64 Hz, square_dark: 1.48 ± 0.91 Hz, *t*_(3)_ = 0.69), maximum firing frequency (circle_dark: 6.11 ± 3.38 Hz, square_dark: 5.10 ± 3.62 Hz, *t*_(3)_ = 1.40) and spatial coherence (circle_dark: 0.61 ± 0.08, square_dark: 0.52 ± 0.14, *t*_(3)_ = 0.97). Spatial information content was slightly lower in square arena (Skaggs, circle_dark: 1.53 ± 0.17, square_dark: 1.04 ± 0.08, *t*_(3)_ = 3.30, *p* < 0.05, mean ± SEM, mean ± SEM, two-tailed *t* test for paired two samples for means, data from representative four cells).

### Claustral Object Cells Fire from the First Exposure to an Environment

**Figure 7:**
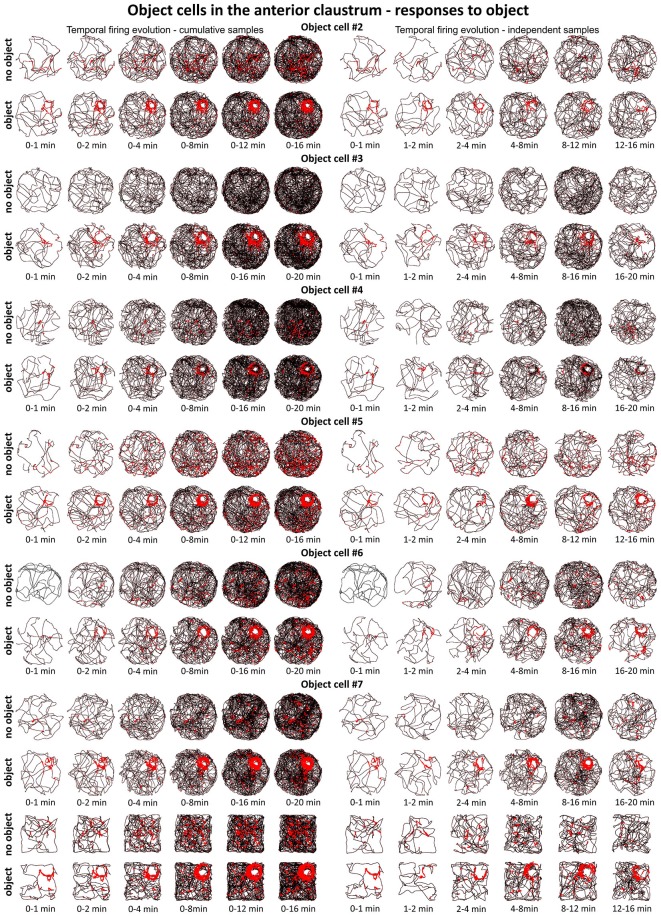
**Temporal evolution of firing of claustral object cells #2–#7 presented in Figures 3 and 4.** For each cell are shown two consecutive recording sessions with and without presentation of the object (glass bottle). For cell #7, additional recordings performed in the square arena are presented. On the left, six samples of cumulative time intervals, and on the right, six samples of independent time intervals are presented in rows for each cell. Sessions were 16 or 20 min long. Object cells responded throughout the whole session from the beginning until the end of the session (cell #3 had an exceptionally low spontaneous firing rate without the object). The visual cue was located in the same place as in Figure 1 in baseline conditions.

Figures 6 and 7 depict the temporal evolution of object cell firing for cumulative samples and independent time-binned samples, demonstrating that object-related activity is present from the first minute of exposure to the environment (irrespective of the environment shape). On Figure 6 object cell #1 from Figure 1 is presented in sessions with environmental manipulations i.e., changed lighting conditions and repositioning of the object on consecutive recording sessions. Figure 7 presents sessions with and without the object for cells #2–#7 and recordings in circular and square arenas for object cell #7.

### Claustral Object Cells can Respond Both to Multiple and New Objects

**Figure 8:**
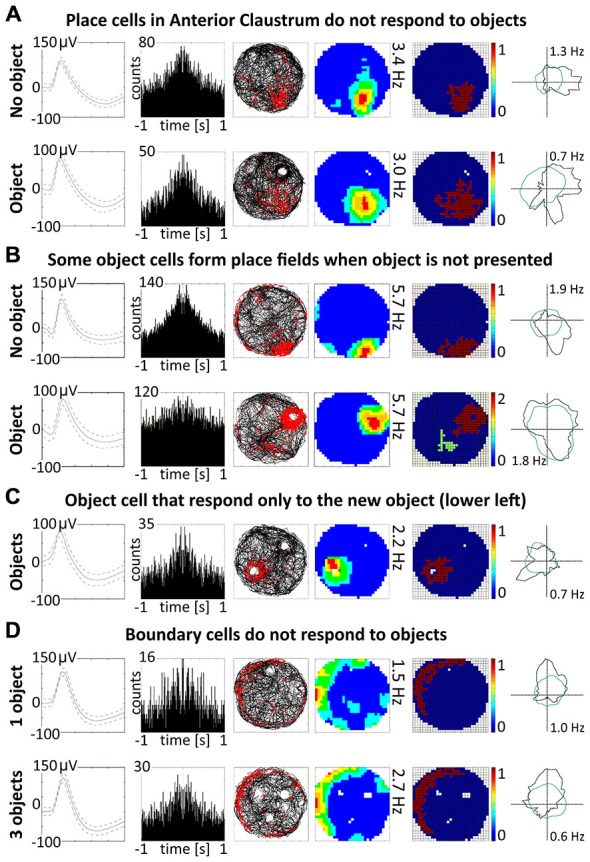
**Other properties of claustral place and object cells. (A)** Place cells do not respond to the objects. An example of a place cell recorded during two subsequent sessions with and without presentation of the object (glass bottle). **(B)** A small proportion of object cells (12.5%) form place fields when recorded without object. **(C)** Two cells were responsive only to the presentation of new object and did not react to known objects. **(D)** Boundary cells do not respond to the objects. For all examples are presented in rows: spike waveform; autocorrelation ± 1000 ms; path of the animal with superimposed firing activity; firing intensity map with a maximum firing frequency, place map and polar plot.

Claustral object cells may respond to the presence of multiple objects simultaneously, and rarely respond specifically to the introduction of new objects. We present some examples of selected cells which responded to two or three objects simultaneously, and cells which responded to all objects with similar firing activity. We tested several combinations: two identical objects, two objects with different features, familiar and new object and three objects. The positions of three objects were changed simultaneously, and the cell responded to the objects on new positions showing that claustral cells can dynamically follow multiple objects (Figure 5). In the majority of trials, object cells responded similarly to new and known (old) objects (Figure 5). In subsequent analyses, we found only two cells that responded only once to the new object, and subsequently stayed silent in all other conditions (Figure 8C).

### A Small Proportion of Object Cells form Place Fields When the Object is not Present in the Environment

A majority of object cells (42 cells, 87.5%) did not form place fields during baseline recordings when an object was not present in the environment. However, six cells (12.5% of all object cells) formed place fields similar to place cells when recorded without the object. When an object was introduced into the environment, those cells redistributed their firing activity adjacent the object, behaving similarly to other object cells (Figure 8B). It is not entirely clear if object cells respond to the object or are activated by sensory inputs occurring when the animal explores the three dimensional elements of content in the environment. However, the existence of object cells that in baseline conditions form place fields suggest that those neurons possess features of spatially-tuned cells. Moreover, some object cells with more robust responses are activated when rat approaches the object or pass it by in the small distance.

### Place, Object and Boundary Cells in the Anterior Claustrum Show Within Session Stability

A particular concern for these cells is whether or not they show the locational boundary-related activity through time, i.e., that they display the necessary degree of inter-temporal stability that characterizes these spatial signals in other brain regions. Although these experiments do not address the issue of between-session stability of these spatial signals, our experiments do address the issue of stability of spatial signals within the recording sessions. We segment the evolution of spatial firing into independent time bins (from 0–1, 1–2, 2–4, 4–8, 8–16 and 16–20 min; see Figures 2, 3, 6, 7). Spatial activity is consistently present within each recording session, across each of the independent time bins, and the spatially-related discharge is consistent in location for claustral place and object cells, and adjacent the wall for boundary cells.

### Theta-Modulated Cells in the Claustrum

**Figure 9:**
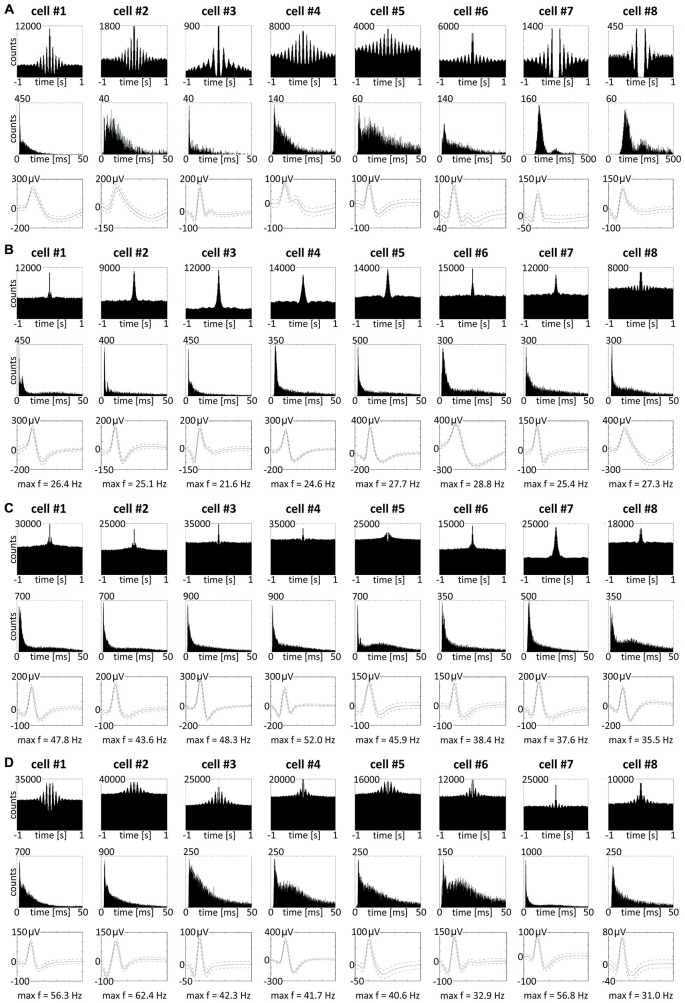
**Theta-modulated and fast-firing bursting cells in rat anterior claustrum. (A)** Claustral neurons with firing activity synchronized in theta frequency range. Examples of bursting theta-modulated cells (cells #1—#6) and late-firing neurons (cells #7—#8). For each cell in columns are shown: autocorrelation ± 1000 ms, interspike interval histogram and spike waveform. **(B–D)** Fast-firing bursting neurons divided into two subclasses: **(B)** cells with maximum firing frequency measured in any place of the open field arena between 20–30 Hz and **(C,D)** cells with maximum firing frequency >30 Hz. Both groups included weakly theta-modulated cells. For each cell in columns are shown: autocorrelation ± 1000 ms, interspike interval histogram, spike waveform and maximum firing frequency.

We also found in the anterior claustrum a small population of theta-modulated cells (24 cells: 2.7% of all recorded units, including cells from other categories). Examples of eight representative cells are shown on Figure 9A (cells #1–#8). We performed autocorrelation analyses (±1000 ms) on these cells, and found modulation of firing activity in the theta frequency range (6–12 Hz). In this group of cells, we observed bursting as well as late, regular firing neurons (Figure 9A; cells #1–#6 vs. #7–#8). Moreover, we observed 37 weakly theta-modulated cells (4.2% of all recorded units, including cells from other categories). Examples of bursting units showing weak theta modulation are shown on Figure 9D. Presence of theta is important because neuronal networks involved in spatial navigation in the rat are strongly entrained by oscillations in theta range (Buzsáki and Moser, 2013). We did not observe any theta-skipping cells (Brandon et al., 2013; Jankowski et al., 2014).

### Fast-Firing Bursting Neurons in Anterior Claustrum

Among recorded units, we also found a group of 96 bursting fast-firing cells (10.9% of all cells) and this category was divided into two classes: 59 bursting cells with a maximum firing frequency 20–30 Hz (6.7%), and 37 bursting cells with a maximum firing frequency >30 Hz (4.2%) at any location in the open field environment (Table 2). Examples of these cells are shown on Figures 9B–D. 32.5% of bursting units with a maximum firing frequency above 30 Hz showed weak theta modulation (Figure 9D), and 8.5% of cells with a maximum firing frequency 20–30 Hz firing were weakly entrained by theta frequency oscillations (Figure 9B; cell #8).

## Discussion

The claustrum has been reasonably well investigated anatomically, but remains largely unexplored behaviorally or electrophysiologically. Our electrophysiological recordings in the freely-moving rat suggest a remarkable pattern of activity in the claustrum, quite unlike that previously described under anesthetized or immobilized conditions. We have found in the anterior claustrum several populations of spatially-responsive cells. One subpopulation of cells remarkably resembles the classic place cells of the hippocampal formation (O’Keefe and Dostrovsky, 1971). These cells are active while the animal is freely moving, show somewhat less-sharply defined place fields during recordings in the dark, and this activity is reinstated when the light is restored. These cells respond to the movement by 180 or 90 degrees of distal cues in the environment. Moreover, these cells do not change their preferred firing location after changes of environment shape. The presence of proximal three-dimensional-objects in the environment does not induce remapping in claustral place cells; these cells do not respond to the object with increased firing activity. However, we have also found in anterior claustrum a distinct group of cells responsive in the vicinity of discrete objects which we classified as “object cells”. These resemble in certain respects the object-responsive cells found in subiculum (Anderson and O’Mara, 2004), lateral entorhinal cortex (Deshmukh and Knierim, 2011; Tsao et al., 2013), anterior cingulate cortex (Weible et al., 2009, 2012) and perirhinal cortex (Burke et al., 2012) because they appear to signal the presence of objects or landmarks. They differ, however, in that their activity dynamically follows the repositioning of the object. They are active when the rat approaches and explores the object; if there is no object in the environment their activity is significantly decreased. We recorded cells responsive to the object in the light and the dark, in environments of different shapes and known as well as novel objects. These cells dynamically follow the object when its position is changed, forming fields of increased firing activity around the new position of the object. Moreover, those cells respond to multiple objects and follow their reconfiguration. Besides place and object cells, we also found a small subset of spatially-tuned cells whose firing activity is selectively increased in the vicinity of geometric borders of the environment. We classified those cells as “boundary cells”, because they share some phenotypic similarities to the boundary vector cells of the subiculum as well as to entorhinal cortex border cells. These cells show only a little directional tuning, similar to the claustral boundary cells described here (Solstad et al., 2008; Lever et al., 2009). Claustral boundary cells preserve their firing properties in light and dark and do not respond to objects (similar to claustral place cells). The fact that claustral boundary cells did not respond to objects suggests that their firing activity was not simply induced by stimulation of somatosensory receptors when animal was exploring near the vertical walls of the arena. On the contrary, object cells, which formed a specific field of increased firing activity around the object, did not respond near the boundaries of the recording arena. This suggests that these two types of cells are probably two functionally distinct populations of neurons. It is not clear if object cells respond to the object as a perceptual entity in some multimodal fashion or if they just simply form a boundary of increased firing activity around any elements that form content of the environment. Object cells are active both in the light and dark, and some of object cells are active when the rat approaches the object, it is possible that those cells are driven conjointly by somatosensory and visual inputs.

The spatial cells in claustrum share some characteristics of other spatial cells, such as those found in the hippocampus and entorhinal cortex (Moser and Moser, 2014). The existence of these cells is remarkable as they are currently unpredicted by any current theory of claustral function or indeed any more general theory of the representation of space within the mammalian brain. The presence of spatial cells in claustrum is highly significant, as the claustrum is a major source of direct projections to widespread anterior cortical regions concerned with memory and action (Wilhite et al., 1986; Park et al., 2012; Zhang et al., 2013). The claustrum also receives widespread cortical and limbic inputs, involving both interoceptive and exteroceptive information (Olson and Graybiel, 1980; Wilhite et al., 1986; Sadowski et al., 1997; Kowiański et al., 1998, 1999, 2001; Zhang et al., 2001; Majak et al., 2002; Park et al., 2012; Zingg et al., 2014). These claustral spatial cells are, therefore, pivotally positioned to influence cortical spatial and action processing directly because of the dense, direct connections of the claustrum with many anterior cortical regions.

Theta rhythm is considered to play critical role in spatial and non-spatial mnemonic functions in brain systems (Buzsáki, 2005). The firing activity we recorded in anterior claustrum (place, boundary and object cells) was not entrained by theta-frequency oscillations, as is found in the hippocampal and entorhinal neuraxes and the anterior thalamic nuclei (Boccara et al., 2010; Tsanov et al., 2011a,b; Buzsáki and Moser, 2013). We also found a small subset of non-spatially-tuned cells whose firing is modulated by the presence of theta, in addition to fast-firing cells showing weak theta modulation. This observation suggests that the claustrum receives theta information (Agarwal et al., 2014) from structures with network activity modulated at theta frequency. Lack of theta modulation in claustral spatially-tuned neurons distinguishes them from those observed in the hippocampal-entorhinal system (Buzsáki and Moser, 2013).

Connectional data suggest that the claustrum has extensive direct and indirect connections with limbic structures involved in spatial navigation (Park et al., 2012; Zhang et al., 2013) and that contain head direction, border, grid and place cells (O’Keefe and Dostrovsky, 1971; Taube et al., 1990; Hafting et al., 2005; Solstad et al., 2008). Moreover, in lateral entorhinal cortex, populations of object cells and object-trace cells have been observed (Tsao et al., 2013). Park et al. (2012) investigated the anatomical connections of the claustrum in Microcebus murinus, and provided possibly the first suggestion that the claustrum may be involved in spatial navigation because of its connectivity. Grasby and Talk (2013) reported that after excitotoxic lesions of anterior claustrum rats had impaired spatial working memory. The cell loss in anterior claustrum resulted in difficulties in finding new but not old positions of the platform in the watermaze, whereas working memory and latent inhibition tasks were unaffected. We recorded in anterior claustrum object cells involved in dynamic mapping of the position of proximal objects in space; thus, our results might partially reinforce the claustral origin of deficits in Grasby and Talk (2013) after cell loss in anterior claustrum. Moreover, claustral place and boundary cells also could be directly involved in spatial working memory in the water maze by dynamical remapping the animal’s position in respect of distal cues. Potentially, all three types of cells can code space, the contents of space and instantaneously react to any reconfiguration of landmarks or objects in space. The fact that rats with anterior claustrum lesions could not find the new platform position in the watermaze supports our hypothesis that claustrum is involved in the dynamic “here and now” representation of the space, while consolidated spatial memory traces are supported by the anterior thalamic-hippocampal-entorhinal system with which claustrum is directly connected (Wilhite et al., 1986; Witter et al., 1988; Aggleton et al., 2010; Park et al., 2012; Jankowski et al., 2013; Zhang et al., 2013). Our results show that claustral object cells rapidly detect objects introduced into the environment, and their activity follows the change in object location; the property’s of the objects seem to be less important for those cells. Claustral object cells did not change response after substituting one object for another of a different shape, color and material. We found just two of 48 object cells that responded only once to the introduction of a completely new object and which did not respond to known objects in any other tested protocol. Claustral place cells also dynamically follow the position of distal landmarks; the location of their place fields changes according to the position of the determining visual cues. Another interesting feature of claustral cells is that place and boundary cells do not respond to objects, whereas a small percentage (12.5%) of object cells can behave like place cells, forming place fields in the absence of proximal objects in the environment. The lack of responsiveness of claustral place cells to objects is an important difference to hippocampal place cells, in which remapping can be controlled by both distal cues and proximal objects (Renaudineau et al., 2007). Manns and Eichenbaum (2009) suggested that objects might be represented as points of interest on the hippocampal cognitive map, useful in remembering encounters with particular objects in specific locations. The question of why some claustral object cells tend to form place fields in the absence of proximal objects in the environment remains open. One possible explanation is that such cells switch between detecting proximal objects to distant objects or cues, but this hypothesis needs to be verified in further studies. Our results show that spatially-tuned cells in rat anterior claustrum dynamically respond to environmental changes and do not form long-lasting traces of activity of previous locations of objects or place fields.

Current theories suggest that the neuronal circuits storing spatial memories are located mainly in the hippocampal formation, which receives spatial information from spatial cells in medial entorhinal cortex and nonspatial information from cells in lateral entorhinal cortex (Buzsáki and Moser, 2013; but see Winocur et al., 2005). Tsao et al. (2013) reported that lateral entorhinal cortex neurons show little spatial modulation when rats run in an empty open-field environments but fire in the vicinity of discrete objects, suggesting that they provide information about the specific content of the spatial environment. Tsao et al. (2013) described two types of cells that react to objects: object cells and object trace cells. Object cells in the anterior claustrum resemble lateral entorhinal cortex object cells because they are active only when the object is present in the environment. Claustral object cells differ from object responsive cells in the inferior temporal cortex of monkeys because they do not form specific object-response associations for selected visual objects (Sakai and Miyashita, 1991; Naya and Suzuki, 2011).

It is still not clear if the claustrum is functionally-homogenous or if its function varies in a regionally-dependent manner across its longitudinal axis. Some studies suggest that anterior part of the rat claustrum shows immunohistochemical staining for parvalbumin and crystalline mu (a putative marker of insular cortex), and that it does not express G protein gamma 2 subunit (Gng2), which is expressed in striatal part of the claustrum (Mathur et al., 2009; Mathur, 2014). Pirone et al. (2012) confirmed that both the Gng2 and the Netrin-G2 proteins show an affinity to the human claustrum and related areas. However, they reported the presence of Gng2 and Netrin-G2 immunoreactive elements in the insular cortex, but not in the putamen, suggesting a possible common ontogeny of the claustrum and insula (Wójcik et al., 2002). Zhang et al. (2013) reported retrogradely labelled cells in the claustrum, which sends very substantial unidirectional projections to the hippocampus; these cells were present at the level of anterior claustrum. Further electrophysiological and optogenetic studies *in vivo* aimed at dissecting claustral neuronal networks are needed to reveal the functional and structural organization of the claustrum.

In summary, our data support the idea that the claustrum dynamically represents both extended space without the body and that it incorporates landmark information from the environment, perhaps acting as a “local action space”. Shima et al. (1996) suggest that the primate claustrum plays a role in non-specific arm-movement motor control. Claustral spatial maps emerge rapidly and are representationally rich. We hypothesize, therefore, that one of the functions of the claustrum may be to process dynamic information about space, boundaries and landmarks, thereby contributing to the moment-to-moment control of behavior. Areas receiving inputs from the claustrum would therefore have action maps of extrapersonal space which they might not otherwise have access to, enabling dynamic responding to environmental cues and objects. We conclude there is another and previously unknown component to the brain’s navigation system, one involving a structure potentially involved in the dynamic modulation of spatial representation and action.

## Materials and Methods

### Rats

Four (4–6 months) male Lister-Hooded rats (B&K, UK) weighing 420–530 g were used. Upon arrival, animals were housed individually and handled by the experimenter daily for a week before being trained in the pellet-chasing task (see below). Rats were food-restricted to 85% of their *ad libitum* body weight and kept in a temperature-controlled laminar airflow unit and maintained on a 12-h light/dark cycle (lights on from 08:00 to 20:00 h).

### Ethics

Experiments were conducted in accordance with European Community directive, 86/609/EC, and the Cruelty to Animals Act, 1876, and was approved by the Comparative Medicine/Bioresources Ethics Committee, Trinity College, Dublin, Ireland, and followed LAST Ireland and international guidelines of good practice. Surgery was conducted under isoflurane anesthesia, an appropriate post-surgery monitoring and analgesia regime was in place, and every effort was made to minimize suffering.

### Behavioral Testing

Experiments were conducted in a circular arena (diameter 96 cm) and square arena (64 × 64 cm). The insides of the arenas were a uniform matt black, and low-level lighting was used during light testing; all lights were extinguished during dark testing. All experiments were conducted during the day between 09:00 and 20:00 h. Session lengths were typically 16–20 min duration. Rats performed a pellet-chasing task during the course of the experiments. During testing, 20 mg food pellets (TestDiet^™^, 5TUL formula, St. Louis, MO, USA) were thrown in the arena at random locations ca. every 20 s. During the weeks of recordings, animals were also allowed 20 g of food daily. The environment is partially curtained with a big visual cue card in a constant location. We left the rat in the environment during the LDL transitions.

The objects used were a collection of glass, wood, plastic, metal or ceramic objects or toys that typically had a base with diameter of about 8 cm and were at least 16 cm high. We used a transparent glass bottle with a textured surface as a baseline object, presented multiple times to all recorded object cells. This object was also used in all protocols for baseline recordings and was presented in following recording sessions: LDL transitions, object repositioning, in first two trials of object-object transposition-object substitution transitions, multiple object recordings, recordings in environments of different shape and recordings with simultaneous presentation of new and the known (old) object as the known object. For object substitution sessions, we used an object of clearly different material, texture, shape and color—a colorful wooden flower. We also used a set of objects presented to the animals only once during the simultaneous presentation of new and known objects as the new object. Usually for this purpose, we used plastic, metal or ceramic objects of a clearly different texture, shape and color (e.g., yellow plastic rocket toy). All objects were cleaned between sessions. In baseline sessions, LDL transitions, object repositioning and baseline recordings of three objects, objects were positioned always in the same positions, which were located in the corners of virtual equilateral triangle inside the circular arena (in baseline sessions in the upper right corner of this triangle). Object-object transposition-object substitution transitions also began from the baseline position but then the object was transposed to the opposite quarter of a circular arena and subsequently substituted in this place with a distinct object. Known and new objects were also presented in opposite quarters of the circular arena (the known object in the right upper quarter). Reconfiguring of the position of simultaneously presented three objects was performed by putting them near the walls in corners of a reversed, larger, equilateral virtual triangle. However, taking into account novelty of observed phenomenon we tested also many other types of objects placed in many positions and configurations in different environmental conditions.

### *In Vivo* Electrophysiology and Surgery

Detailed descriptions of the surgical protocol and recording techniques can be found elsewhere (Tsanov et al., 2011a; Wang et al., 2012; Jankowski et al., 2014). Briefly, rats were implanted with bundles of eight tetrodes of ø 25 μm platinum–iridium wires with impedance 150–350 kΩ (California Fine Wire Ltd., CA, USA; see Figure 1 for histological verification) or eight ø 17 μm platinum—iridium wire tetrodes with impedance 350–650 kΩ (California Fine Wire Ltd., CA, USA) supported and separated by polyimide tubes (MicroLumen, Oldsmar, FL, USA; see Figure 4 for histological verification). Tetrodes were mounted onto small driveable microdrives (Axona Ltd., UK) and at following coordinates targeted at the claustrum: 2.8 mm anterior to bregma, 3.5 mm lateral to the midline, 3.5 mm below brain surface and at angle of about 13 degrees. Rats were allowed at least 1 week of recovery post-surgery. Recordings were performed using Axona recording system and software DacqUSB (Axona Ltd., UK). Tetrodes were lowered slowly through the brain (maximal rates 25–50 μm/day), typically over a period of weeks to prevent tissue damage and to ensure successful claustral electrode targeting and penetration. Based on the daily record of the electrode position and post-mortem histological verification, each recording could be located along the tetrode trace. The recording sessions took place in curtained arenas located in the center of the test room, which contained large visual cue made salient to allow the animals to orient themselves in the environment. An example of a claustral object cell is provided in Supplementary Video 1.

### Recording and Statistical Analysis

Standard statistical testing used Matlab scripts and Axona software. Signals were amplified between 3000 and 12000 and bandpass filtered between 380 Hz and 7 kHz for single-unit detection. To maximize unit separation, only waveforms of sufficient amplitude (of at least three times noise threshold) were acquired. The amplitude of each spike was measured as the difference between the positive peak and first negative peak before the positive peak, if present, or zero. The height was measured as the difference between the peak to the minimum value of the spike waveform. The width of the spikes was determined as the distance in microseconds beyond which the waveform drops below 25% of its peak value. Candidate waveforms were then discriminated offline using graphical cluster-cutting software (Tint, Axona Ltd., UK). Unit identification involved several criteria. First, neurons had to be active in all conditions and had to present the same waveform characteristics (amplitude, height, and duration) in those conditions. Furthermore, units had to demonstrate a clean refractory period (>2 ms) in the inter-spike interval (ISI) histogram. Once well-defined neuronal signals were isolated and rats explored the arena sufficiently (rats had to explore at least 90% of the open field in either session to be included in analysis to allow reliable calculation of spatial characteristics), recording commenced. In total, 874 well-isolated units recorded in four rats were assigned to the claustrum after post-mortem histological verification. To select animals for analysis we set following criterion: histologically-verified electrodes should be placed in anterior claustrum, electrode tracks were localized predominantly in this central portion of anterior claustrum. In each of four rats, we recorded 414, 340, 78 and 42 cells, respectively.

### Spatial Analyses

Firing rate maps allow for visual inspection of the preferred areas of firing for the neurons (i.e., place fields). They were constructed by dividing the number of spikes that occurred in specific pixel coordinates by the total trial time the animal spent in that coordinate. This produced maps depicting the place fields of each cell in Hertz. The pixel map is converted into array of square bins 3 cm on a side. Autoscaled color-coded firing rate maps were then created to visualize firing rate distributions (Muller and Kubie, 1987). In such maps, pixels in which no spikes occurred during the whole session are displayed as blue. The highest firing rate is coded as red, and intermediate rates are shown as orange, yellow, green, and cyan pixels from high to low. We used multiple indices to analyze the spatial properties of the claustrum place, boundary and object cells firing [namely spatial coherence and spatial information content expressed in bits per spike (Skaggs et al., 1993, 1996; Gothard et al., 1996)]. High information content per spike signifies that a single spike is a strong predictor of the location of the rat. A firing field was defined as a set of at least nine contiguous pixels with firing rate above zero. A place field was identified if nine neighboring pixels (sharing a side) were above 20% of the peak firing rate (Hollup et al., 2001; Brun et al., 2002). Place field size was represented by number of pixels. The spatial selectivity of a firing field (ratio of maximal signal to noise) was calculated by dividing the firing rate of the cell in the bin with the maximum average rate by its mean firing rate over the entire apparatus (Skaggs et al., 1996). Spatial coherence consists of a spatial autocorrelation of the place field map and measures the extent to which the firing rate in a particular bin is predicted by the average rate of the eight surrounding bins. Thus, high positive values result if the rate for each bin could be better predicted given the firing frequency of the neighboring location (Muller and Kubie, 1989; Quirk et al., 1990; Sharp and Green, 1994). Mean frequency is the total number of spikes divided by the total recording time and is expressed in Hz. Exploration was assessed by comparing the occupancy of bins and the number of visits per bin between the two recording conditions. Similar to other studies (Leutgeb et al., 2007; Fenton et al., 2010), cells were classified as spatially-tuned cells (i.e., place, boundary and object cells) if their spatial firing patterns were location specific (spatial coherence >0.25; spatial information content >0.5 bits/action potential; mean firing rate >0.25 Hz). Claustral cells were subjected to similar analyses to allow comparisons of properties between these described for the first time cells.

### Histological Analyses

On completion of the recording studies, the rats received an overdose of anesthetic (1.5 g of urethane (Sigma-Aldrich) dissolved in 4.5 ml water) and were then perfused intracardially with 250 ml of 0.1 M phosphate-buffered saline (PBS) at room temperature followed by 350 ml of 4% paraformaldehyde in 0.1 M PBS at ~4°C, after which the brains were removed and placed in 4% paraformaldehyde (for at least 72 h). Brains were blocked, placed on a freezing platform, and 20 μm coronal sections were cut with a sledge microtome (Leica 1400). All sections were taken through the anterior claustrum. Brain slices were mounted directly onto gelatine-subbed slides, and then allowed to dry overnight before staining with cresyl violet, a Nissl stain.

Recording positions were determined by calculating the distance above the deepest electrode position, and calculating the distance below the first penetration into the tissue. The electrodes sometimes caused tissue distortion, which was compensated for in the position calculations. Positions of recorded cells were estimated as follows: theoretical positions of electrodes tips and anterior claustrum borderlines were estimated by reference to Paxinos and Watson (2004) and reconstructed histological specimens; tissue distortion was adjusted for when making the position calculations. The position of the electrodes below the brain surface was determined for each recording session and expressed in μm, thereby allowing estimates of each cell position to be proportionally derived.

## Author Contributions

MJ and SMOM conceived these experiments; MJ conducted experiments and analyzed data; MJ and SMOM co-wrote the paper.

## Conflict of Interest Statement

The authors declare that the research was conducted in the absence of any commercial or financial relationships that could be construed as a potential conflict of interest.

## Supplementary Material

Online data archive: the data from which this paper was generated are archived at Dryad and may be accessed via http://www.tara.tcd.ie/handle/2262/74729

The Supplementary Video for this article can be found online at: http://journal.frontiersin.org/article/10.3389/fnbeh.2015.00250

Supplementary Video 1 **Raw data sample showing a representative well-discriminated object cell recorded in anterior claustrum while the rat is engaged in pellet chasing task in a circular arena.** One animation of a whole 16 min session is presented in fast motion mode (recording accelerated 20×). The unit corresponds to unit #7 of Figure 5.
